# Data-driven targets for reducing the global burden of TB

**DOI:** 10.5588/ijtldopen.25.0014

**Published:** 2025-06-13

**Authors:** C.R. Horsburgh, Helen E Jenkins, L. Martinez, L.F. White

**Affiliations:** ^1^ Department of Epidemiology, Boston University School of Public Health Boston MA USA;; ^2^ Department of Global Health, Boston University School of Public Health Boston MA USA;; ^3^ Department of Biostatistics, Boston University School of Public Health, Boston MA USA.

**Keywords:** tuberculosis, TB prevalence, TB reduction, TB incidence

## Abstract

**BACKGROUND:**

The proportion of persons with infectious TB that need to be cured to reduce prevalence is an important but not well characterized target for TB control.

**METHODS:**

We compared infectious TB prevalence from countries with two population-based surveys since 2000, accounting for persons receiving curative treatment and those dying or undergoing natural recovery. Annual incidence was estimated as the proportion of prevalence that, when applied to each year over the interval between the two surveys, yielded the observed second survey prevalence. We then determined the relationship between the proportion of people with TB cured and the change in prevalence in each of the years covered by the surveys.

**RESULTS:**

Achieving a decline in prevalence required curing at least 20% of those with infectious TB. None of the countries studied reached the 11% annual decline in prevalence required to yield the END TB goal of a 90% decrease in prevalence over 20 years; this would require diagnosing and curing 35-40% of people with prevalent TB each year.

**CONCLUSIONS:**

These results provide targets for achieving the goal of a 90% reduction in TB and indicate that active case finding will be required to reach these targets.

TB caused an estimated 1.25 million deaths in 2023, the largest cause of global mortality from a single infectious agent.^1^ Reducing the global burden of TB has been a WHO priority since 1994, yet only incremental progress has been made.^1^ Reaching the goal of TB elimination – defined as less than 1 person with TB per million population – within 40 years will require a sustained decrease in incidence of 16% per year.^2^ In 1998, a modeling study predicted that a country needed to identify only 70% as many people with TB as were estimated to have acquired smear-positive TB in that year (‘incident TB’) and cure 85% of them annually to reduce the TB burden by 11% per year.^3^ However, few TB programs were able to reach this target and even those that did failed to achieve substantial reductions.^4^ Moreover, measuring incidence has proven challenging, so it was often unclear whether targets had been reached.^5,6^

We hypothesized that data-driven targets for TB program performance might be identified using the results from population-based TB prevalence surveys. Since 2000, procedures for performing national population-based TB prevalence surveys have been standardized and such surveys have been implemented in 32 countries.^7^ We reviewed the results from countries reporting two sequential prevalence surveys to model the relationship between the proportion of persons with TB that were successfully treated (‘cured’) and the change in TB prevalence, allowing us to identify targets for TB program performance based on prevalence rather than incidence.

## METHODS

### Comparison of successive surveys.

All countries with two successive national prevalence surveys since 2000 were considered for inclusion (prior to 2000 national prevalence survey methodology was not standardized).^7^ Eight countries conducted two population-based national TB prevalence surveys in that interval: Bangladesh, China, Cambodia, Indonesia, Myanmar, the Philippines, Thailand and Vietnam. Participation in the 2006 Thailand study was less than 40%,^7^ and the first Bangladesh survey collected sputum from all participants regardless of symptoms but radiographs and symptoms were not reported, while the second survey only collected sputum from persons with symptoms or a positive radiograph; thus there was no basis for comparing the two surveys.^8^ In addition, because the DOTS program was not implemented equally in all provinces between 1990 and 2000,^9^ the most recent China survey was compared only to the one in 2000. After these exclusions, six countries with two comparable prevalence surveys were included in our analysis. Where appropriate, we adjusted for methodologic differences between the two within-country surveys to improve comparability. In cases where specific publications comparing the two surveys and adjusting for these differences were available, we used the adjusted estimates.

### Modeling strategy

**Figure 1.**
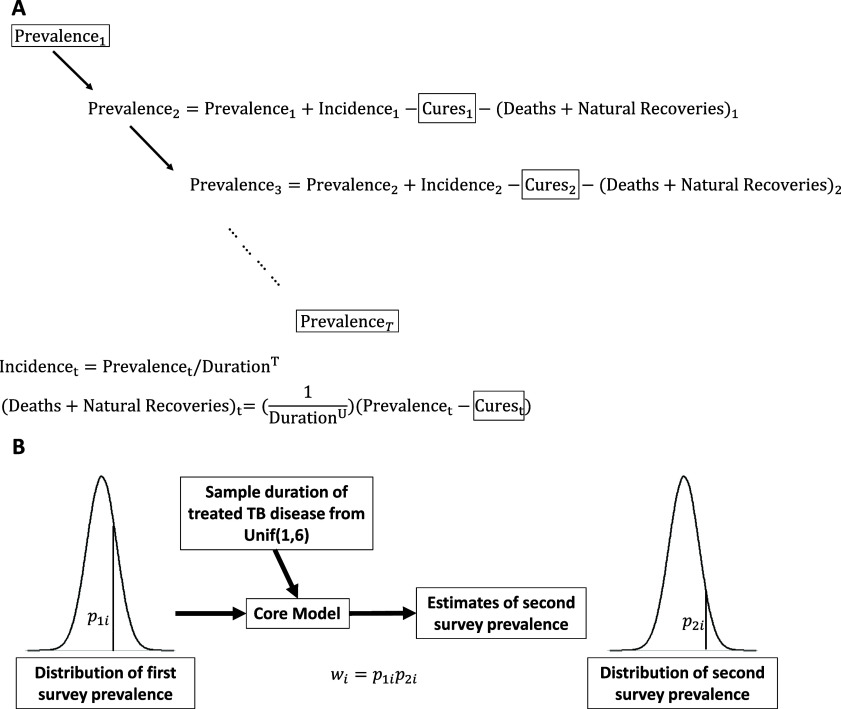
Diagrammatic representation of model. **A:** depicts the core model simulation. Parameters in boxes are data that are reported in prevalence surveys or by programs to WHO. Cure data are reported cures/treatment completions at each time point (as reported to the WHO). **B:** depicts the Sample Importance Resampling approach to estimation of duration of the infectious period and incidence of new (or recurrent) infectiousness. We assume that the first prevalence value follows a normal distribution and sample from this; we sample a value for the duration of untreated disease from a uniform distribution to run the core model and obtain an estimated second prevalence survey value. We then calculate a weight that is the product of the density value from the first and second prevalence survey values, based on the normal distribution. These weights are normalized and then used to get an empirical posterior distribution of the duration of untreated disease. These estimates of duration and prevalence allow us to estimate incidence.

We built a model to derive TB duration and incidence each year between the two prevalence surveys, assuming that the ratio of prevalence to incidence was constant over the studied time period (Figure 1). The model assumes that people with infectious TB *either* receive curative treatment in each year *or* do not receive curative treatment during that year, progressing to death or self-cure. We calculated prevalence in year t, $P_{t}$ as a function of the prior year's prevalence, number treated, number who died or underwent natural recovery and the incidence in the prior year, as follows: $P_{t}=P_{t-1}-C_{t-1}-D_{t-1}+I_{t-1}$ where $C_{t}$ is the number of persons with TB treated and cured in that year, $D_{t}$ is the number of persons with TB dying or undergoing natural recovery in that year, and I_t-1_ is the incidence in the prior year. We estimate $D_{t}=\left(\frac{1}{\delta^{U}}\right)\left(P_{t}-C_{t}\right)$, where $\delta^{U}$ is the duration of time spent by people not receiving treatment from first becoming infectious until spontaneous resolution of infectiousness (‘natural recovery’) or death from untreated TB. Here $\left(P_{t}-C_{t}\right)$ describes the number untreated in year t and $\frac{1}{\delta^{U}}$ is the expected proportion of these individuals who are not treated in a one-year period. We assigned a value of 5 years to the average duration of those who die or undergo natural recovery, $\delta^{U}$. To explore the importance of this assumption, we varied our estimates of TB duration among people that were untreated in each survey, from three to seven years, based on published estimates.^10-14^ Finally, we rewrite the incidence, $I_{t-1}$, as the ratio of prevalence to duration of treated disease, i.e. $P_{t}=P_{t-1}-C_{t-1}-\left(\frac{1}{\delta^{U}}\right)\left(P_{t-1}-C_{t-1}\right)+P_{t-1}/\delta^{T}$, where $\delta^{T}$ is the duration of infectiousness prior to treatment initiation for those who initiate treatment.

We obtained the number of people notified with TB and the proportion cured (or completed treatment) in each country during the years comprising the two surveys from the WHO Global TB database.^15^ We combined prevalence per 100,000 individuals with the estimated population^16^ when the prevalence surveys were conducted to estimate the total number of persons with prevalent TB and multiplied this by the proportion reported to have been cured (or completed treatment) to derive the number cured. We used a sample importance resampling approach to estimate the posterior distribution of the duration of the infectious period in persons eventually receiving curative treatment, under the assumption that infectiousness ceased when treatment was initiated (for details see supplement).

All data were obtained from publicly available sources; therefore, the work was exempt from ethical review. All code is available (https://github.com/forsbee/TB-duration-estimation).

## RESULTS

### Estimation of the change in prevalence between surveys

We obtained estimates for each of the two TB surveys for each country as follows below (and as summarized in Table 1). For exact calculations for adjustments not reported by survey investigators see Supplementary Data.

**Table 1.** TB prevalence and trends over time in countries having two successive surveys (upper and lower 95% confidence intervals in parentheses).

Country	1st Survey Year(s)	Prevalence from first survey per 100,000	2^nd^ Survey Year(s)	Prevalence from second survey per 100,000	Time between surveys (years)	Overall percent change in prevalence between 1^st^ and 2^nd^ surveys	Average annual change between surveys+
China	2000	178 (163–195)	2010	116 (101–132)	10	-34.8	-4.2%
Myanmar	2009-10	955 (878–1033)*	2017-18	468 (391–546)	9	-51.0	-7.6%
Cambodia	2002	1497 (1238–1808)	2011	831 (707–977)	9	-44.5	-6.3%
Vietnam	2007	510 (441–580)^+^	2017	322 (260–399)	10	-36.9	-4.5%
Philippines	2007	1049 (906–1191)*	2016	1159 (1016–1301)	9	+10.5	+1.1%
Indonesia	2004	506 (321–692)*	2013-14	759 (590–961)	10	+50.0	+4.1%

* First survey results for Myanmar were calculated using the reported decline in prevalence of 51%^21^. First survey results for Indonesia were calculated using the estimated increase of 50% prevalence between surveys (see results). First survey results for Philippines were calculated using the reported increase of 10.5% prevalence between surveys.^7^ First survey prevalence for Vietnam was back-calculated from the second survey using independently reported annual rate of change in prevalence of 4.5%.^18^

+ We assume that the relationship between the first and second prevalence surveys is described by $p_{2}=r^{T}p_{1}$, where r and T are the annual rate of change and number of years between the surveys, respectively.

### Vietnam

The 2007 survey results are summarized in the WHO compendium.^7^ The survey found a prevalence of bacteriologically confirmed TB among those 15 years of age and older to be 307 (249–366) per 100,000. The second survey in 2017-2018 found a prevalence of bacteriologically confirmed TB among those 15 years of age and older of 322 (260–399) per 100,000.^17^ However, the methods used in the two surveys differed substantially. A subsequent publication adjusted the results so that the two surveys could be directly compared and found that prevalence of culture-positive TB declined by an average annual decline of 4.5% between the two surveys, which we use in our calculations.^18^ Therefore, to make the results comparable, we back-calculated from 2017-18 to 2007 to identify the starting prevalence for bacteriologically positive TB that yielded an annual decline of 4.5%. This resulted in an estimate for the first survey year of 510 (441–580) per 100,000.

### Indonesia

The 2004 survey results were reported in 2007.^19^ That survey used symptom screening followed by microscopy and culture in all those with a positive symptom screen; chest radiography was not performed. The survey found a prevalence of 120 (79–161) smear-positive persons with TB per 100,000 adults. We compare this to the 2013-2014 survey result of 257 (210–303) persons with smear-positive TB, only 70% of whom were symptomatic. Thus, per the criteria of the 2004 survey, only 180 persons would have been detected. Therefore, the change over the 9.5-year period was 180/120, or an increase of 50%, yielding an average annual increase of 4.1%. The 2013-2014 survey found a prevalence of bacteriologically confirmed TB among those 15 years of age and older of 759 (590–961) per 100,000.^7^ To make the results comparable between the two surveys, we applied the increase of 50% to the estimate of bacteriologically positive TB from 2013-2014, which gave an estimate for the first survey year of 506 (321–692) per 100,000.

### Philippines

The 2007 survey results revealed a prevalence of bacteriologically confirmed TB among persons 10 years of age or greater of 660 (530–800) per 100,000 and for the total population, the corresponding estimates were 576 (515-640).^7^ The 2016 survey found a prevalence of bacteriologically confirmed TB among those 15 years of age and older to be 1159 (1016-1301) per 100,000. The methods used in the two surveys differed slightly; when these differences were adjusted for, TB prevalence increased by 10.5% over the 10-year interval, an average annual increase of 1.1%.^7^ Therefore, to make the results comparable, we back-calculated from 2016 to 2007 to identify the starting prevalence for bacteriologically positive TB that yielded an annual increase of 1.1%. This gave an estimate for the first survey year of 1049 (906–1191).

### China

The 2010 survey in China revealed a prevalence of bacteriologically confirmed TB among persons 15 years of age or greater of 116 (101–132) per 100,000 persons.^9^ Analysis comparing bacteriologically positive prevalence in the 2000 and 2010 surveys adjusting for differences in study design showed a prevalence of bacteriologically positive TB in 2000 of 178 (163–195) per 100,000^9^; this is a decrease of 34.8% between 2000 and 2010, an average annual decrease of 4.2%.^19^

### Cambodia

The 2010-2011 survey in Cambodia revealed a prevalence of bacteriologically confirmed TB among persons 15 years of age or greater of 831 (707–977) per 100,000 persons.^7^ An analysis comparing the results of the 2002 and 2011 surveys adjusting for differences in the methodologies revealed that the prevalence of bacteriologically-positive TB in 2002 was 1497 (1238–1808).^20^ Thus, there was a 44.5% reduction in prevalence over the 9-year period, an average annual reduction of 6.3%.

### Myanmar

The 2017-2018 survey in Myanmar revealed a prevalence of bacteriologically confirmed TB among persons 15 years of age or greater of 468 (391–546) per 100,000 persons.^21^ When the results of the 2009-2010 survey were adjusted to be comparable to those of the 2017-2018 survey, it was noted that there had been a 51% reduction between the two surveys, translating to an average annual decrease of 7.6%.^21^ Therefore, the prevalence of bacteriologically-positive TB in 2009-10 is estimated to have been 955 (878–1033).

### Average duration of TB (incidence as a proportion of prevalence)

In Table 2 we show the duration and corresponding incidence that produce the observed changes in prevalence between the first and second survey under the modeled conditions for each country (assuming a five-year duration of time from initiation of infectiousness to death or natural recovery). The effect of varying assumed duration of time from first becoming infectious to spontaneous resolution of infectiousness or death from untreated TB untreated disease on average duration of time from first becoming infectious to initiation of curative therapy, by country is shown in Supplementary Data Figure S1.

**Table 2.** Duration of infectiousness estimates in persons eventually receiving curative treatment and the corresponding incidence of infectious TB under the assumption that the duration of time from initiation of infectiousness to death or natural recovery of those not cured was 5 years.

Country	Average duration of the infectious period in persons eventually receiving curative treatment (years)	Lower confidence limit	Upper confidence limit	Average incidence of infectious TB as a proportion of prevalence	Lower confidence limit	Upper confidence limit
Cambodia	3.30	3.01	3.59	0.303	0.279	0.332
China	2.48	2.40	2.57	0.403	0.389	0.418
Indonesia	2.54	2.18	2.87	0.393	0.348	0.459
Myanmar	2.68	2.57	2.79	0.373	0.358	0.389
Philippines	2.83	2.68	2.99	0.353	0.334	0.373
Vietnam	2.84	2.67	3.02	0.352	0.331	0.375

### Estimated annual prevalence

**Figure 2.**
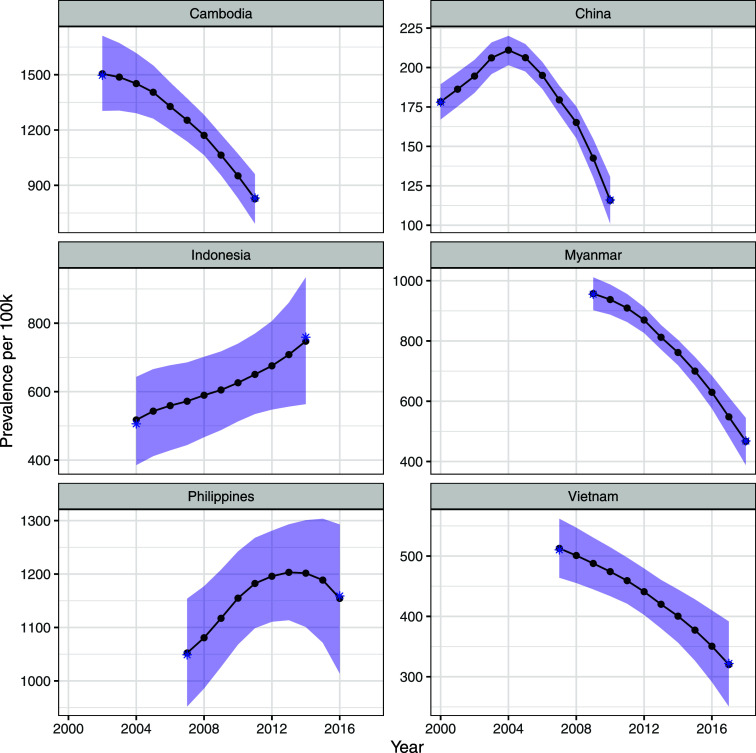
Trajectories of infectious TB prevalence between the first and second surveys with confidence intervals (shaded).

We show the estimated annual prevalence incorporating the uncertainty in the first prevalence value and the uncertainty in the duration estimate in Figure 2. There was very little effect on estimated annual prevalence when varying time from first becoming infectious to spontaneous resolution of infectiousness or death from untreated TB untreated disease from 3 to 7 years (Supplementary Data Figure S2).

### Proportion of persons with prevalent TB cured in relation to related change in prevalence

**Figure 3.**
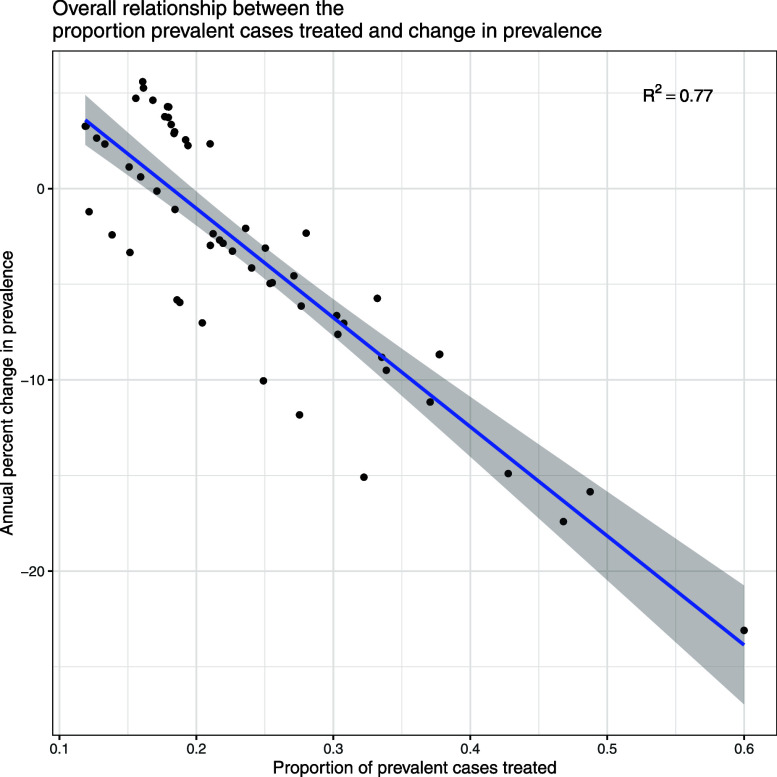
Overall relationship between the proportion of people with infectious TB cured and change in prevalence. Each point represents a single year on one of the countries studied (for individual country plots see Supplementary Data Figure S3).

As seen in Figure 3, when less than 20% of persons with prevalent TB were cured in a year, prevalence increased; when more than 20% of persons with prevalent TB were cured in a year, prevalence decreased. Curing 27% of prevalent TB cases was associated with a 5% reduction in prevalence; curing 35% of prevalent TB cases was associated with a 10% reduction in prevalence, and curing 53% of prevalent TB cases was associated with a 20% reduction in prevalence. There was some variability between countries, as shown in Supplementary Data Figure S3, but the general trends are similar.

## DISCUSSION

We found that TB prevalence decreased substantially in four of the six countries with two successive surveys of comparable data quality. The countries that reduced their TB burden achieved average reductions of from 1–8.1% per year. Our model indicates that, on average, 20% or more of people with TB must be diagnosed and cured each year to begin to reduce prevalence. These findings reflect the current understanding that persons with TB who have not presented for clinical care are an important reservoir of infectious TB, and that such persons need to be identified and cured in order to reduce the force of TB infection.^22–24^ This suggests why earlier targets of identifying 70% as many people with TB as were estimated to have acquired smear-positive TB in that year and curing 85% of them did not lead to reductions in the global TB burden.

Our model focuses on the average duration of time that people with TB are infectious, because reducing the pool of infectious persons by shortening the time such persons are circulating in the community is an important output of efforts to reduce the TB burden. To do this, we separated the model into two compartments, those that were removed from the infectious pool by curative treatment, and those that were not treated but rather underwent natural recovery or died with TB. This dichotomization allows the model to address shortening of the overall average duration of time spent in the infectious pool by identifying and treating infectious cases while maintaining a constant average duration of time spent in the pool by persons not identified and cured. Those who undergo natural recovery could subsequently re-enter the infectious pool as ‘incident’ disease; thus, our definition of incidence (defined as prevalence/duration) is not directly comparable to that generally applied.^5,6^

Our results provide guidance for setting targets for TB elimination. As shown in Figure 3, curing an average of 20% or more of prevalent disease was associated with reduction in the size of the infectious pool. There was some variability by country, but overall this average appeared to be representative of the countries studied. Thus, reducing the prevalence of TB will require setting higher targets of the proportion of people with TB diagnosed and cured and demonstrating that these targets have been achieved. Finding and curing more than 20% of persons with TB will be difficult, but it can be achieved with robust active case finding. Three studies of community wide screening and treatment exceeded this threshold and achieved impressive reductions in prevalence.^25–27^

Our results are limited in that the HIV burden in the countries we examined was low. Among included countries, Myanmar had the highest proportion of HIV among persons with TB at 8%. Thus, our results may not be applicable to countries where the burden of HIV is more substantial. To date, no high-HIV burden countries have completed a second TB prevalence survey; when second surveys are completed in such countries, expanding our model with data from paired surveys and HIV treatment parameters will be important. Our adjustments of the first survey results to make them comparable to those of the second survey (when this was not performed by the investigators) may also have introduced inaccuracies, but we believe that these were small and did not have critical effects on the model outputs. Our calculations also may undercount people cured in the private sector. If this were the case, the actual proportion of people leaving the pool each year could be greater than we have measured. In addition, we did not account for changes in the characteristics of the patient risk factors or TB program performance during the interval. However, we believe that these were not large and were unlikely to have introduced substantial bias.

These results can be helpful for establishing targets for diagnosing and curing TB that do not rely on estimates of TB incidence. For countries that are similar in the characteristics of their TB epidemic to those we studied, our model could provide a way for TB programs to set targets: after performing a prevalence survey, the amount of decline in prevalence desired could be selected and the target number of persons with TB needing to be cured could be estimated. At the conclusion of a year, the proportion cured could be applied to predict current prevalence and new targets could be set.

Our results demonstrate that current levels of case finding were able to identify and cure enough people with TB to reduce disease prevalence in only four of the six countries studied, and in the countries that achieved reductions, these reductions were unlikely to lead to TB elimination within 20 years. Thus, expanded case finding will be needed to increase the number of people diagnosed, treated and cured and to make substantial progress in reducing the global TB burden.

## Supplementary Material


