# Supplementary material for: Data-driven targets for reducing the global burden of TB
Source: IJTLD Open. 2025 Jun 13;2(6):352–8. doi: 10.5588/ijtldopen.25.0014 (PMC12168725; doi:10.5588/ijtldopen.25.0014)

Supplement to “Data-driven targets for reducing the global tuberculosis burden”

Contents:

1. Sample importance resampling approach to estimating the duration of the infectious period in persons eventually receiving curative treatment (under the assumption that infectiousness ceases when treatment was initiated).
2. Table S1. Model results for each country under the assumption that the average time from initiation of infectiousness to death or natural recovery among persons not receiving curative treatment is 5 years.
3. Figure S1. Effect of varying assumed duration of time from first becoming infectious to spontaneous resolution of infectiousness or death from untreated TB on average duration of time from first becoming infectious to initiation of curative therapy, by country.
4. Figure S2. Trajectories of tuberculosis prevalence between the first and second survey, stratified by differing assumptions of the duration of infectiousness among persons not receiving curative treatment.
5. Figure S3. Country level relationship between the proportion of persons with prevalent disease treated in a year and the change in prevalence over that year.

Sample importance resampling approach to estimating the duration of the infectious period in persons eventually receiving curative treatment. Our approach accounts for uncertainty in the first and second prevalence survey estimates by assuming these are normally distributed according to the estimate and 95% confidence interval reported in the prevalence surveys. We assume a uniform prior for the infectious period among persons receiving curative treatment ranges between 1 and 6 years. To determine the posterior of the infectious period among persons receiving curative treatment, we use the following empirical approach. We repeat the following procedure 100,000 times to obtain 100,000 weights. We sample a first prevalence survey value from a normal distribution based on the reported first survey prevalence and values for the infectious period from a uniform(1,6) distribution. We then estimate the second prevalence survey value using the formula outlined in the methods section of the main text and shown in Figure 1A. Using the assumption that the first and second prevalence survey estimates are normally distributed, we calculate the joint probability of the calculated second prevalence survey value along with the sampled first prevalence survey value. We normalize these 100,000 joint probabilities to obtain weights that sum to one. We then sample 10,000 times from the 100,000 uniformly sampled duration of infectiousness values with replacement, using these weights as sampling probabilities. This provides an empirical distribution of the infectiousness period for persons receiving curative treatment. Using these values, along with the corresponding first survey prevalence values, we use the model to calculate 10,000 vectors of annual prevalence. The model structure is shown in Figure 1. With these estimated annual prevalence values, we calculate the proportion of prevalent cases treated as the ratio of annual WHO reported cures to the annual number of prevalent cases. We also determine the rate of change in annual prevalence as the difference between successive prevalence estimates divided by the annual prevalence, i.e. $\Delta Prev = (P_t - P_{t-1})/P_t$.

Table S1. Model results for each country under the assumption that the average time from initiation of infectiousness to death or natural recovery is 5 years.

Year	Untreated duration (years)	Country	Prevalent cases	Population	Treatment starts	Proportion treated	Prev per 100k	Prev LCL	Prev UCL	Change in prevalence	%Change in Prevalence
2000	5	China	2248	1,262,645,000	NA	NA	178.3	167.1	189.4	NA	NA
2001	5	China	2365	1,271,850,000	423	0.18	186.2	175.3	197.0	8.0	4.3
2002	5	China	2486	1,280,400,000	447	0.18	194.6	184.0	204.9	8.3	4.3
2003	5	China	2650	1,288,400,000	426	0.16	206.1	195.8	215.9	11.5	5.6
2004	5	China	2729	1,296,075,000	573	0.21	211.0	201.5	220.0	4.9	2.3
2005	5	China	2681	1,303,720,000	751	0.28	206.2	197.5	214.8	-4.8	-2.3
2006	5	China	2547	1,311,020,000	846	0.33	195.0	186.4	203.5	-11.2	-5.7
2007	5	China	2354	1,317,885,000	888	0.38	179.4	170.5	188.4	-15.6	-8.7
2008	5	China	2174	1,324,655,000	821	0.38	165.2	155.1	175.3	-14.3	-8.7
2009	5	China	1881	1,331,260,000	917	0.49	142.6	130.4	154.7	-22.6	-15.9
2010	5	China	1529	1,337,705,000	917	0.60	115.8	101.0	130.6	-26.7	-23.1
2002	5	Cambodia	188	12,561,779	NA	NA	1505.1	1303.9	1710.7	NA	NA
2003	5	Cambodia	189	12,787,710	23	0.12	1487.0	1305.6	1671.4	-18.0	-1.2
2004	5	Cambodia	188	13,016,371	26	0.14	1451.8	1291.3	1616.8	-35.2	-2.4
2005	5	Cambodia	185	13,246,583	28	0.15	1404.9	1262.1	1549.7	-47.0	-3.3
2006	5	Cambodia	178	13,477,779	33	0.19	1327.7	1201.2	1457.4	-77.2	-5.8

2007	5	Cambodia	170	13,714,791	32	0.19	1253.1	1138.1	1370.7	-74.6	-6.0
2008	5	Cambodia	162	13,943,888	33	0.20	1170.9	1064.1	1281.3	-82.2	-7.0
2009	5	Cambodia	149	14,155,740	37	0.25	1063.9	954.1	1175.4	-107.0	-10.1
2010	5	Cambodia	134	14,363,532	37	0.28	951.4	830.1	1069.7	-112.5	-11.8
2011	5	Cambodia	118	14,573,885	38	0.32	826.7	691.7	959.6	-124.7	-15.1
2004	5	Indonesia	1143	225,938,595	NA	NA	517.4	385.4	643.2	NA	NA
2005	5	Indonesia	1214	228,805,144	189	0.16	543.0	411.7	665.8	25.6	4.7
2006	5	Indonesia	1263	231,797,427	232	0.18	559.1	428.6	677.1	16.1	2.9
2007	5	Indonesia	1305	234,858,289	253	0.19	572.0	444.0	685.4	12.9	2.3
2008	5	Indonesia	1358	237,936,543	250	0.18	589.5	467.0	701.8	17.5	3.0
2009	5	Indonesia	1405	240,981,299	270	0.19	604.9	487.9	717.6	15.4	2.6
2010	5	Indonesia	1465	244,016,173	266	0.18	625.9	513.1	740.2	21.0	3.4
2011	5	Indonesia	1532	247,099,697	271	0.18	650.3	534.6	769.6	24.4	3.8
2012	5	Indonesia	1599	250,222,695	287	0.18	675.4	547.6	806.1	25.1	3.7
2013	5	Indonesia	1683	253,275,918	283	0.17	708.1	556.3	859.9	32.7	4.6
2014	5	Indonesia	1779	256,229,761	287	0.16	747.4	563.6	934.1	39.3	5.3
2009	5	Myanmar	468	49,015,836	NA	NA	957.1	902.2	1011.4	NA	NA
2010	5	Myanmar	462	49,390,988	109	0.24	937.6	887.7	987.6	-19.5	-2.1
2011	5	Myanmar	451	49,794,522	113	0.25	909.3	863.0	955.9	-28.3	-3.1
2012	5	Myanmar	435	50,218,185	118	0.27	869.6	826.5	912.6	-39.7	-4.6
2013	5	Myanmar	410	50,648,334	126	0.31	812.4	771.9	853.0	-57.2	-7.0
2014	5	Myanmar	387	51,072,436	117	0.30	761.8	719.5	803.6	-50.6	-6.6
2015	5	Myanmar	358	51,483,949	120	0.34	700.0	652.8	746.7	-61.8	-8.8
2016	5	Myanmar	324	51,892,349	120	0.37	629.8	575.5	684.7	-70.3	-11.2
2017	5	Myanmar	283	52,288,341	121	0.43	548.1	482.8	613.6	-81.7	-14.9
2018	5	Myanmar	241	52,666,014	113	0.47	466.8	388.0	544.6	-81.3	-17.4
2007	5	Philippines	939	89,561,377	NA	NA	1052.2	952.0	1154.0	NA	NA

2008	5	Philippines	983	91,252,326	125	0.13	1080.7	985.8	1177.3	28.5	2.6
2009	5	Philippines	1035	92,946,951	123	0.12	1117.1	1026.3	1208.1	36.4	3.3
2010	5	Philippines	1089	94,636,700	130	0.12	1154.8	1068.0	1242.4	37.7	3.3
2011	5	Philippines	1134	96,337,913	151	0.13	1182.4	1098.7	1267.9	27.5	2.3
2012	5	Philippines	1167	98,032,317	176	0.15	1195.9	1110.7	1281.1	13.5	1.1
2013	5	Philippines	1193	99,700,107	190	0.16	1203.2	1113.6	1293.2	7.3	0.6
2014	5	Philippines	1210	101,325,201	207	0.17	1201.6	1100.9	1300.9	-1.6	-0.1
2015	5	Philippines	1215	103,031,365	224	0.18	1188.7	1071.5	1303.6	-12.9	-1.1
2016	5	Philippines	1199	104,875,266	252	0.21	1154.4	1013.7	1293.0	-34.2	-3.0
2007	5	Vietnam	433	84,762,269	NA	NA	512.7	464.1	561.9	NA	NA
2008	5	Vietnam	426	85,597,241	89.6	0.21	500.9	455.3	547.0	-11.8	-2.4
2009	5	Vietnam	419	86,482,923	89.9	0.21	487.8	444.9	530.6	-13.1	-2.7
2010	5	Vietnam	412	87,411,012	89.1	0.22	474.2	433.7	514.6	-13.6	-2.9
2011	5	Vietnam	402	88,349,117	89.6	0.22	459.2	421.1	497.5	-15.0	-3.3
2012	5	Vietnam	390	89,301,326	91.9	0.24	440.9	402.6	479.4	-18.3	-4.2
2013	5	Vietnam	375	90,267,739	92.9	0.25	420.1	380.2	460.5	-20.8	-5.0
2014	5	Vietnam	360	91,235,504	89.4	0.25	400.4	356.7	444.7	-19.7	-4.9
2015	5	Vietnam	341	92,191,398	91.3	0.27	377.2	326.7	428.2	-23.2	-6.1
2016	5	Vietnam	319	93,126,529	92.8	0.29	350.5	291.2	410.0	-26.7	-7.6
2017	5	Vietnam	292	94,033,048	93.9	0.32	320.1	250.6	391.6	-30.4	-9.5

Figure S1. Effect of varying assumed duration of time from first becoming infectious to spontaneous resolution of infectiousness or death from untreated TB on average duration of time from first becoming infectious to initiation of curative therapy, by country.

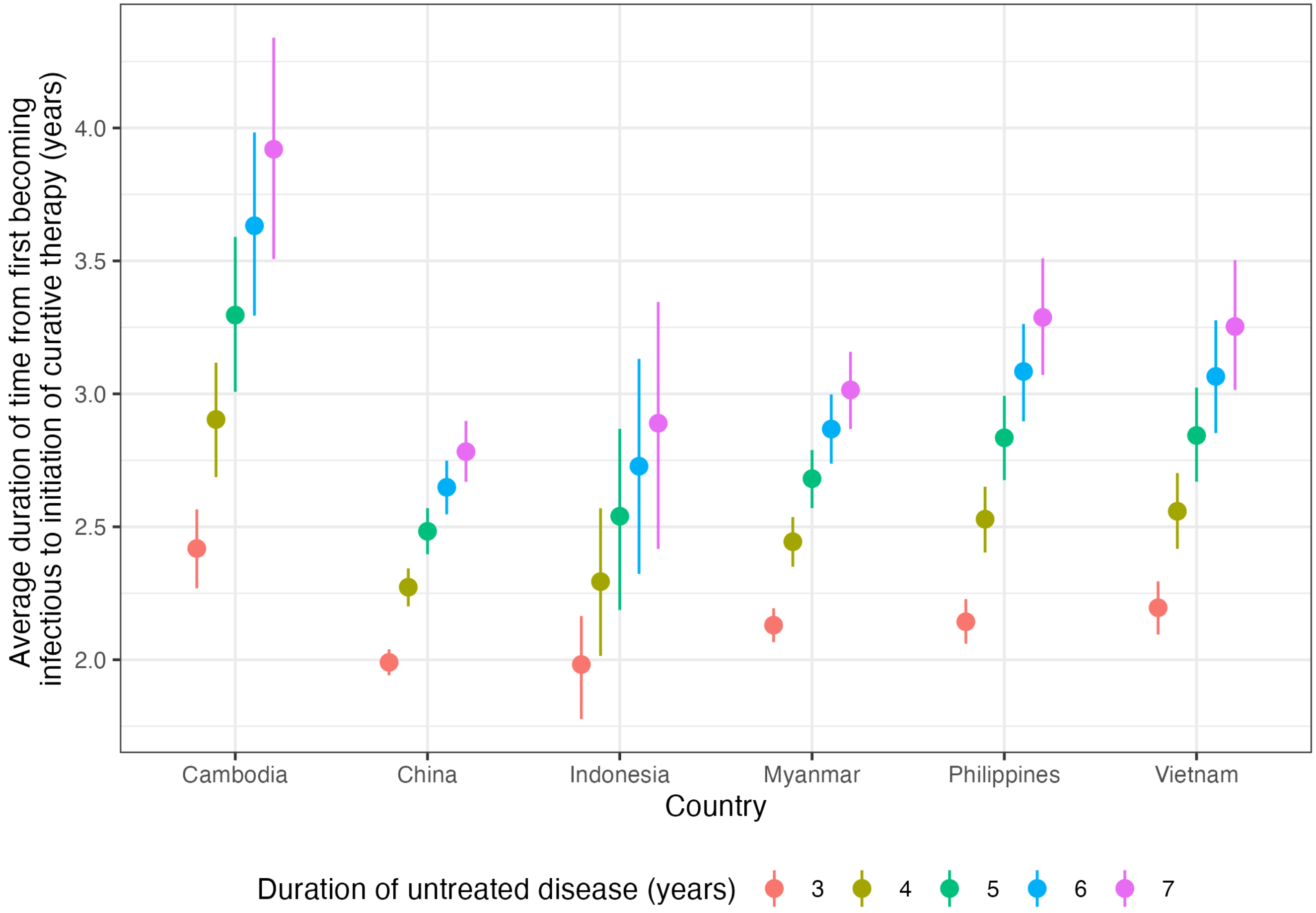

Figure S2. Trajectories of tuberculosis prevalence between the first and second survey, stratified by differing assumptions of the duration of infectiousness among persons not receiving curative therapy.

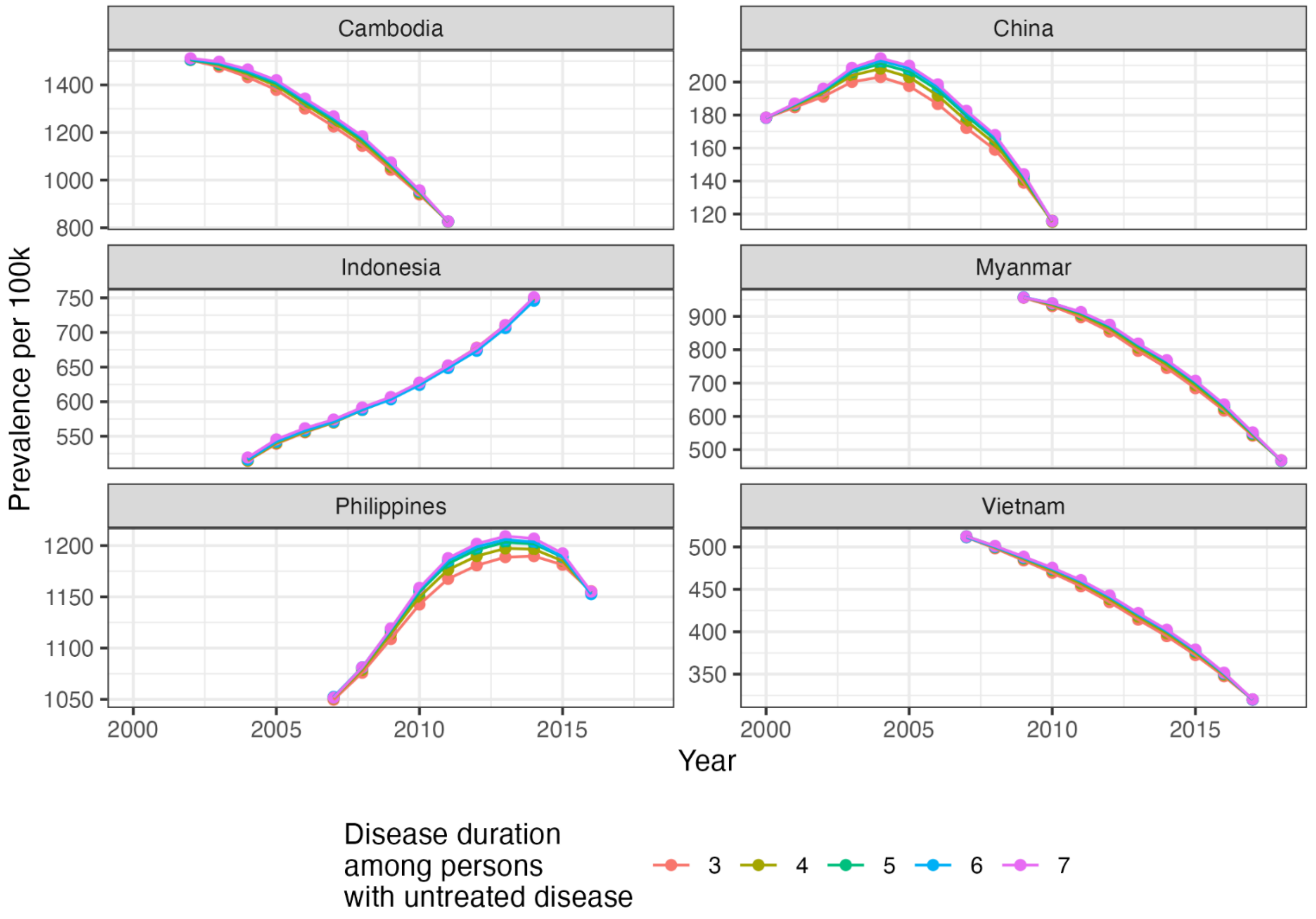

Figure S3. Country level relationship between the proportion of persons with prevalent disease treated in a year and the change in prevalence over that year.

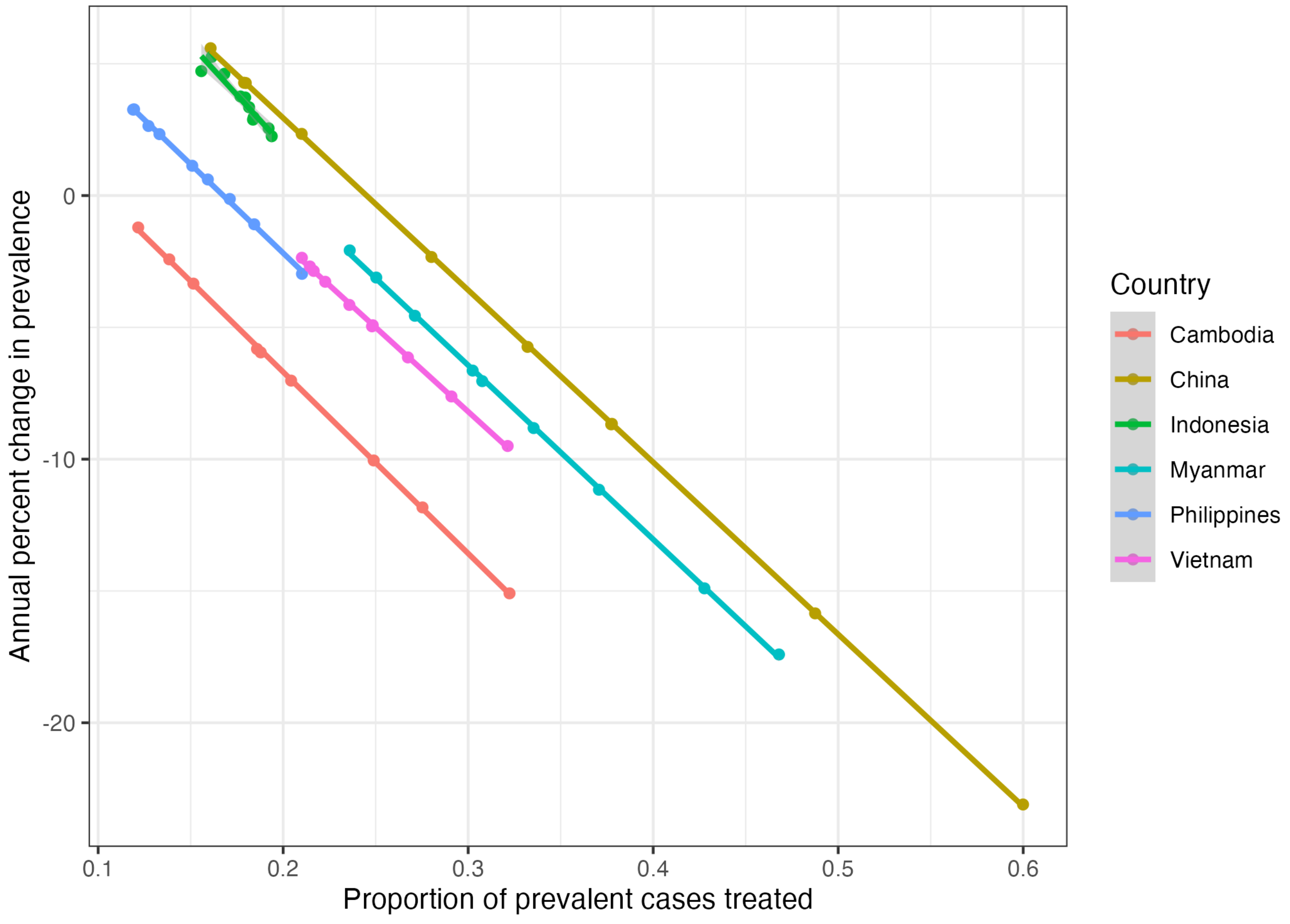

Supplement: Supplementary file 1 [file ijtldopen25-0014_supplementarydata1.pdf]
